# The Epigenomics of Pituitary Adenoma

**DOI:** 10.3389/fendo.2019.00290

**Published:** 2019-05-14

**Authors:** Blake M. Hauser, Ashley Lau, Saksham Gupta, Wenya Linda Bi, Ian F. Dunn

**Affiliations:** ^1^Center for Skull Base and Pituitary Surgery, Department of Neurosurgery, Brigham and Women's Hospital and Harvard Medical School, Boston, MA, United States; ^2^Department of Neurosurgery, University of Oklahoma Health Sciences Center, Oklahoma City, OK, United States

**Keywords:** pituitary tumor, pituitary adenoma, epigenetics, precision medicine, endocrine surgery, epigenome

## Abstract

**Background:** The vast majority of pituitary tumors are benign and behave accordingly; however, a fraction are invasive and are more aggressive, with a very small fraction being frankly malignant. The cellular pathways that drive transformation in pituitary neoplasms are poorly characterized, and current classification methods are not reliable correlates of clinical behavior. Novel techniques in epigenetics, the study of alterations in gene expression without changes to the genetic code, provide a new dimension to characterize tumors, and may hold implications for prognostication and management.

**Methods:** We conducted a review of primary epigenetic studies of pituitary tumors with a focus on histone modification, DNA methylation, and transcript modification.

**Results:** High levels of methylation have been identified in invasive and large pituitary tumors. DNA methyltransferase overexpression has been detected in pituitary tumors, especially in macroadenomas. Methylation differences at CpG sites in promoter regions may distinguish several types of tumors from normal pituitary tissue. Histone modifications have been linked to increased p53 expression and longer progression-free survival in pituitary tumors; sirtuins are expressed at higher values in GH-expressing compared to nonfunctional adenomas and correlate inversely with size in somatotrophs. Upregulation in citrullinating enzymes may be an early pathogenic marker of prolactinomas. Numerous genes involved with cell growth and signaling show altered methylation status for pituitary tumors, including cell cycle regulators, components of signal transduction pathways, apoptotic regulators, and pituitary developmental signals.

**Conclusions:** The limited clinical predictive capacity of the current pituitary tumor classification system suggests that tumor subclasses likely remain to be discovered. Ongoing epigenetic studies could provide a basis for adding methylation and/or acetylation screening to standard pituitary tumor workups. Identifying robust correlations between tumor epigenetics and corresponding histological, radiographic, and clinical course information could ultimately inform clinical decision-making.

## Introduction

Pituitary tumors constitute at least 15% of intracranial neoplasms (1–4). The anterior pituitary is composed of several hormone-producing cell types, including corticotrophs, somatotrophs, lactotrophs, mammosomatotrophs, thyrotrophs, and gonadotrophs, all of which can give rise to tumors, leading to the heterogeneous group of neoplasms encompassed by the diagnosis of pituitary adenomas (5, 6). Recent work suggests that the term “pituitary tumor” may be more appropriate than “pituitary adenoma,” but “adenoma” has been used in this review in some instances to accurately reflect findings reported in the literature (7). These tumors can be functional—producing hormones that reflect their lineage with concordant systemic effects—or nonfunctional, producing systemic sequelae through compromised pituitary function. Each general group can produce symptoms by offending any of a number of adjacent anatomical structures. These groups and individual tumors can have a wide range of clinical behaviors, from benign to highly invasive. Their long-term behavior and response to therapy are not reliably predicted by current classification methods.

**Figure 1:**
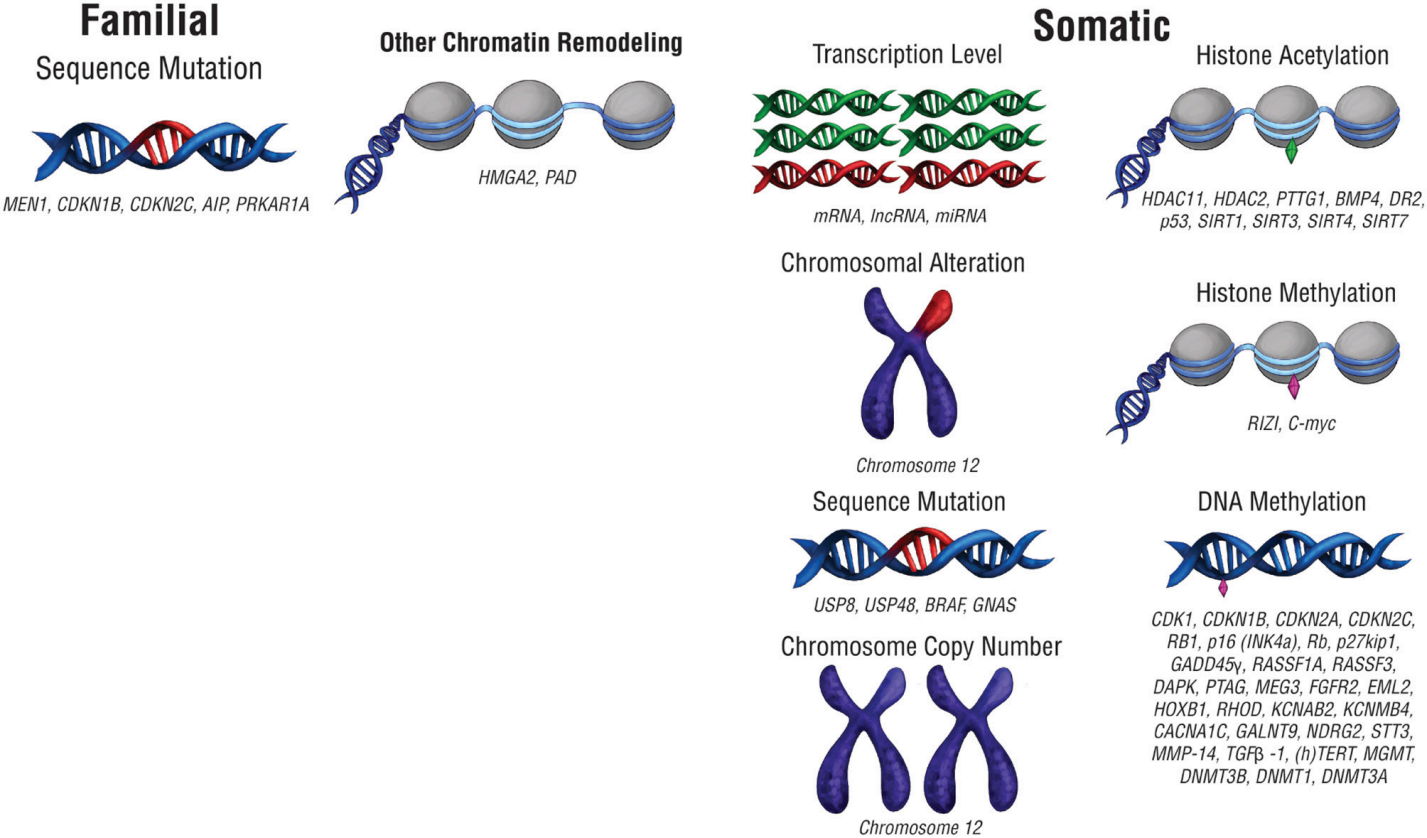
Pre-translational modifications that affect gene expression. Modifications that affect gene expression can occur at any point along the path from packaged DNA to protein expression. Alterations to chromosome structure or changes in chromosome copy number can affect expression at a chromosomal level. Epigenetic chromatin modifications, including histone acetylation and histone methylation, can also alter gene expression levels. At the DNA level, mutations in DNA sequence or DNA methylation can change the nature of the genes expressed as well as their level of expression. RNA transcript copy number also affects protein expression.

The biological underpinnings of pituitary tumors have been investigated to predict and manage them with more precision. The accumulation of genetic mutations confers downstream oncogenic changes such as sustained proliferation, invasion, angiogenesis, growth suppression evasion, and cell death resistance (8). These mutations can consist of changes to the DNA sequence, as well as chromosomal alterations and copy number changes (Figure 1; Table 1). Large scale genomic sequencing has revealed several mutations in subtypes of pituitary adenomas (9–18). However, on the whole, mutations that drive oncogenesis are sparse across pituitary tumors. Consequently, non-mutational sources of gene expression alteration in pituitary tumors are undergoing investigation.

**Table 1:** Genetic, regulatory, and epigenetic mutations.

**Genetic**	**RNA interference**	**Epigenetic**
Sequence mutation	Long non-coding RNA (lncRNA)	DNA methylation
Chromosome alteration	microRNA (miRNA)	Histone acetylation
Chromosome copy number change		Histone methylation Histone citrullination

*Changes in gene expression can result from changes in DNA sequence, RNA expression as mediated by RNA interference, or epigenetic regulation. The changes in each of these categories discussed in this review are outlined below*.

Epigenetics—the study of alterations in gene expression without changes to DNA sequence—provides an alternative avenue of tumorigenesis and disease characterization (Figure 1). DNA methylation by DNA methyltransferases (DMNTs), amongst other enzymes, typically silences gene expression by reducing the access of transcriptional machinery to methylated segments of DNA (Table 1). Changes to histone placement can also affect DNA transcription. Histone deacetylases (HDACs) and sirtuins modulate histone acetylation, which generally improves transcriptional access to surrounding DNA, while histone methyltransferases like RIZI alter methylation, which either improves or restricts transcriptional access depending on the methylation site (Table 1). Histone citrullination can also affect chromatin expression, and it can be mediated by peptidylarginine deaminase (PAD) enzymes (Table 1).

Epigenetic changes can also alter mRNA transcript levels, resulting in either upregulation or downregulation of gene expression. Changes in messenger RNA (mRNA) transcript levels can occur as part of oncogenic transformation (Figure 1; Table 1). Alterations in mRNA expression can modulate downstream changes in protein expression levels, which in turn drive cellular function. Additionally, differential expression of long non-coding RNA (lncRNA) and microRNA (miRNA) can result in or accompany oncogenesis (Table 1).

Incorporating epigenetics into tumor classification schemes for other types of cancer has improved clinical reliability. In breast cancer, DNA hypermethylation of promoter CpG islands corresponds to the presence of certain hormonal receptors as well as clinical tumor progression (19). Similarly, promoter methylation in glioblastoma correlates with response to therapy (20), and DNA methylation-based classification schemes have shown utility in tumor subclassification and prognosis in meningioma (21). For pituitary tumors, subclassification is a more complicated problem given that the multiple cell types present in the pituitary can give rise to tumors with varied secretory properties. Additionally, with the exception of metastasis, criteria for pituitary tumor malignancy remain unclear. Recent studies profiling epigenetic changes in pituitary tumors have shed new insights into the classification of pituitary tumors and may possibly augment prediction of clinical behavior.

## Current Classification Schemes

The World Health Organization (WHO) classification draws upon pituitary adenohypophyseal cell lineage to categorize tumors into acidophilic, corticotroph, and gonadotroph subtypes based on transcription factor and hormone expression (22, 23). The absence of hormones and transcription factors defines a null cell adenoma. Subtypes with a propensity to exhibit invasiveness, rapid growth, recurrence, and resistance are categorized as clinically aggressive. Terms such as “aggressive,” “invasive,” and “large” are sometimes used interchangeably in the literature, even though these terms refer to distinct features of pituitary tumors. This review maintains the same terminology accompanying the discussed finding from the cited literature. Clinically aggressive tumors often have high mitotic activity and Ki-67 expression but are not defined by a single biomarker (24). Invasive tumors are loosely defined by a combination of clinical, radiological, and histopathological findings, and do not necessarily imply clinical aggressiveness in terms of disease control or recurrence risk (24). Aggressive tumors are defined by clinical characteristics, and primarily reflect a tumor's rate of recurrence (25). Invasion of the dura can be seen in up to 45.5% of pituitary adenomas (26). Combining multiple modes of information to classify tumors likely provides more accurate prognostic information (27). Novel biomarkers may facilitate the division of pituitary tumors into more clinically useful categories.

## Genomic Alterations

Large-scale genomic studies to identify molecular alterations have been thorough, but pituitary tumors display relatively few genetic aberrations compared to other tumor types and cancers. As a result, pituitary tumor genetic information has limited potential to inform the course of treatment for the numerous pituitary tumors without these identified genetic aberrations. Genome-wide association studies (GWAS) have been used to identify genetic markers associated with pituitary tumor development. GWAS has revealed common variants (10p12.31, 10q21.1, and 13q12.13) that are associated with sporadic pituitary tumors (17).

Recurrent genetic mutations have been identified in small subsets of pituitary tumors. The first category of tumors with recurrent genetic mutations are those that arise due to familial syndromes (Table 2) which include McCune-Albright syndrome, multiple endocrine neoplasia types 1 and 4 (MEN1 and MEN4), familial isolated pituitary adenomas (FIPA), and Carney complex (28). Interestingly, only a small percentage of sporadic pituitary tumors harbor mutations in the genes implicated in familial pituitary tumor disorders [*MEN1*, Cyclin Dependent Kinase Inhibitor 1B *(CDKN1B)*, Cyclin Dependent Kinase Inhibitor 2C *(CDKN2C)*, Aryl-Hydrocarbon Receptor Interacting Protein (*AIP)*, and Protein Kinase cAMP-Dependent Type 1 Regulatory Subunit Alpha *(PRKAR1A)* (9–11)]. Select somatic genetic alterations have been identified in several subtypes of adenomas, including high mobility group A 2 (*HMGA2*) amplification via focal amplification or abnormalities of chromosome 12 in prolactinomas (12), Ubiquitin Specific Peptidase 8 (*USP8)*, Ubiquitin Specific Peptidase 48 *(USP48)*, and *BRAF* in corticotroph adenomas (13, 15, 29), and activating mutations in *GNAS* in GH-secreting pituitary adenomas (14, 16). Chromosome arm-level copy-number alterations also recur within a subset of pituitary tumors, the majority of which are functional macroadenomas (18). In some cases, familial mutations and chromosome abnormalities have been associated with larger tumor size. Genetic associations offer limited utility beyond distinguishing tumor subtype, which may indicate that epigenetic regulation plays a role in the clinical course of pituitary tumors.

**Table 2:** Familial and somatic mutations associated with pituitary tumors.

**Familial syndrome**	**Gene affected (Germline)**
Multiple endocrine neoplasia type 1	*MEN1, CDKN1B, CDKN2C*
Multiple endocrine neoplasia type 4	*CDKN1B*
Familial isolated pituitary adenomas	*AIP*
Carney complex	*PRKAR1A*
McCune-Albright	*GNAS*
**Tumor subtype**	**Gene affected**
Prolactinoma	*HMGA2* (a)
Corticotroph	*USP8, USP48, BRAF*
GH-secreting	*GNAS*

*Familial syndromes are listed in the top part of the table, and subtype-specific somatic alterations and their mechanisms are listed in the bottom portion of the table. a, amplification; all other genes are mutated*.

## Transcriptional Profiling

The distinct gene expression profiles of pituitary tumors correlate to some extent with hormone expression status. Additionally, gene expression profiles may have some predictive value with respect to clinical aggressiveness (18). Tumor classification systems with a molecular basis often yield more insight into tumor origin, tumor behavior, and probable clinical outcomes than purely histological approaches (21, 30, 31). The idea that gene expression signatures may provide insights about tumor behavior and outcome has motivated transcriptomic studies in pituitary tumors to gain a better understanding of how signatures correlate with tumor properties and patient outcomes. These studies also permit the evaluation of the effect of genetic mutations on a protein level, which can improve the clinical utility of tumor genetic information.

Subtypes of pituitary tumors express distinct transcriptional profiles from each other and from normal pituitary gland tissue as assessed by gene microarrays and RNA-Seq. Given that transcription profile differences correlate with tumor presence and subtype, it is possible that they also offer a molecular approach to improving classification schemes. Relative to normal pituitary tissue, pituitary tumors have differentially expressed mRNA transcripts (32–37), lncRNA transcripts (36, 38), and miRNA transcripts (39–42). Notably, investigations have found that two miRNAs (miR196a-2 and miR-212) which target HMGA transcripts can be deregulated in all tumor types (43–47). Changes in expression profile also manifest in different subclasses of tumors (48–50). In particular, deregulation of miR-183 in prolactinomas has been associated with clinical aggressiveness (51).

However, large-scale transcriptome analyses often produce gene expression results that conflict with the findings in other studies. Heterogeneity within tumor samples, a small patient sample that fails to capture a representative selection of tumor samples, and different experimental conditions may contribute to divergent study results. Furthermore, one potential shortcoming of transcriptome studies is the use of predetermined histological or radiographic categories to partition gene expression results. This approach can identify gene expression differences representative of each tumor type or subclass but precludes identification of novel classes within the tumor population that are independent of histological characteristics. The disparate clinical trajectories of pituitary tumor subtypes suggest that there are likely to be subclasses with varying degrees of invasiveness and aggressiveness that remain to be discovered. However, no system for classifying pituitary tumors accurately correlates genetic markers with clinical outcomes, so tumor genetic information still has limited clinical utility.

## Epigenetic Modifications

Given the centrality of gene expression changes and the relative dearth of genetic abnormalities in pituitary tumors, epigenetic modifications have received considerable attention.

**Figure 2:**
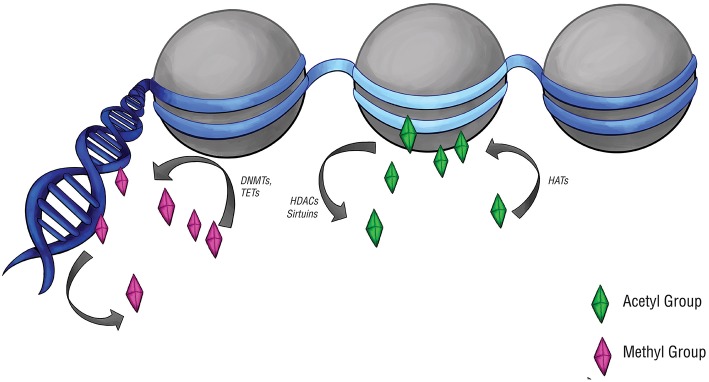
Epigenetic modifications and regulators. Epigenetic modifications are carried out by a set of specialized enzymes that act directly on DNA or on the histones around which chromatin is wrapped. Histone acetyltransferases (HATs) place acetyl groups on chromatin, while histone deacetylases (HDACs) and sirtuins do the opposite. Acetylation of histones typically upregulates gene expression, denoted here by the light blue region of DNA. DNA methyltransferases (DNMTs) and ten-eleven translocation enzymes (TETs) can alter the level of DNA or histone methylation, which typically downregulates gene expression. This is denoted by the navy-blue regions of DNA.

DNA methylation was examined (Figure 2), particularly at CpG islands in gene promoters, where methylation often correlates with gene silencing (52). Approximately two-thirds of reference epigenomes have been found to contain quiescent signatures, whereas only 5% of genomes contain promoter and enhancer signatures (53). The “histone code” hypothesis, which states that certain patterns of post-translational modifications on histone tails can function as signals in gene regulatory processes (54) (Figure 2), has also led to a small number of studies that assay histone modifications in pituitary tumors.

### DNA Methylation

Methylation of gene promoters is frequently deregulated in tumors (55), and appears to be a common mechanism for gene inactivation in pituitary tumors. Given the simplicity of detecting DNA methylation at targeted locations within the genome, clinically meaningful epigenetic findings could be implemented rapidly to guide treatment plan development.

DNA methyltransferase (DNMT) enzymes catalyze methylation at CpG dinucleotides, with DNMT3A and DNMT3B serving as the *de novo* methyltransferases, and DNMT1 as the maintenance methyltransferase. Ten-eleven translocation (TET) enzymes may also participate in regulating methylation as removers of methylation modifications (56). Early observations that classic oncogene and tumor suppressor mutations were absent in pituitary tumors led to the realization that promoter methylation changes constituted an alternative mechanism by which causative genes could be deregulated. Numerous genes involved with cell growth and signaling show altered methylation status, including cell cycle regulators [Cyclin Dependent Kinase 1 (*CDK1)* (57), *CDKN1B* (58), Cyclin Dependent Kinase Inhibitor 2A *(CDKN2A)* (59), *CDKN2C* (59, 60), Retinoblastoma Transcriptional Corepressor 1 *(RB1)* (58, 61), CDKN2A protein (p16^INK4a^) (58), Retinoblastoma (*Rb)* (62), CDKN1B protein (p27kip1) (63), Growth Arrest and DNA Damage 45γ *(GADD45*γ*)* (64, 65)]; components of signal transduction pathways [Ras Associated Domain Family Member 1A (*RASSF1A)* (66) and Ras Associated Domain Family Member 3 *(RASSF3)*]; apoptotic regulators [Death-Associated Protein Kinase (*DAPK)* (67) and Pituitary Tumor Apoptosis Gene *(PTAG)* (68)]; developmental gene Maternally Expressed 3 *(MEG3)* (69); and the growth factor signaling component Fibroblast Growth Factor Receptor 2 *(FGFR2)* (70).

#### DNA Methylation Enzymes

High levels of methylation may be associated with clinically aggressive behavior in pituitary tumors (Table 3). DNMT1 and DNMT3A overexpression has been detected in pituitary tumors (77). Both were significantly associated with more aggressive tumors, with DNMT1 levels also significantly higher in macroadenomas. Relatively higher levels of expression of DNMT3B has also been found in pituitary tumors in comparison to normal tissue with no difference in DNMT1 and DNMT3A expression (71). It is possible that the transfer of methyl groups will also result in regions of DNA being hypomethylated and therefore expressed at a higher level. As DNA hypomethylation has also shown some association with cancerous behavior, high levels of DNMT expression could theoretically increase the risk of malignancy through hypomethylation mechanism as well (79).

**Table 3:** Altered regulation of epigenetic modifiers in aggressive, invasive, or large, and functional tumors.

	**Pituitary tumor**	**Functional**	**Aggressive, invasive, or large**
Upregulated	DNMT3B (71), HDAC11 (72)	HDAC2 (73), RIZI (74), PAD (75), SIRT1 (76), SIRT3 (76), SIRT4 (76), SIRT7 (76)	DNMT1 (77), DNMT3A (77)
Downregulated	HMGA2 (78)		SIRT1 (76), SIRT3 (76)

*DNA modifiers (in red) and histone modifiers (in blue) shown. (Gene names italicized, protein names non-italicized)*.

#### CpG Methylation

Genome-wide methylome studies have found methylation differences at promoter region CpG sites to distinguish several types of adenomas from normal pituitary tissue (80). A subset of genes are hypermethylated in nonfunctional adenomas as well as growth hormone (GH) and prolactin (PRL) secreting adenomas. However, only Echinoderm Microtubule Associated Protein Like 2 *(EML2)*, Homeobox B1 *(HOXB1)*, and Rho-Related GTP-Binding Protein *(RHOD)*, also demonstrate a corresponding decrease in expression, suggesting that the degree of promoter methylation may not always translate to actual changes in gene expression. *HOXB1* has been identified as a tumor suppressor gene in glioma (81), and *RHOD* may affect cytoskeletal reorganization and transportation (82).

Variations in methylation may also exist at CpG sites across the genome, including intergenic sites and gene body regions (83, 84). Nonfunctional tumors have displayed global hypermethylation relative to hormonally active tumors (84), particularly GH (83). Genes involved in ion channel signaling, including Voltage-Gated Potassium Channel Subunit Beta-2 *(KCNAB2)*, Calcium-Activated Potassium Channel Subunit Beta-4 *(KCNMB4)*, and Calcium Voltage-Gated Channel Subunit Alpha1 C *(CACNA1C)*, can be hypermethylated in nonfunctional tumors (83). However, expression does not consistently correlate with methylation at promoter CpG sites, so it is unclear to what extent such epigenetic changes affect phenotype. Hypomethylated CpGs are significantly more common in invasive NF pituitary adenomas than hypermethylated CpGs (84). One differentially methylated site was associated with Polypeptide N-acetylgalactosaminyltransferase 9 *(GALNT9)*, and its expression was downregulated significantly in the invasive tumors. A number of other differentially methylated sites correspond to genes involved in cell adhesion, indicating a possible mechanism by which methylation changes influence tumor phenotype. Characteristic methylation patterns can also be associated with GH-secreting, ACTH-secreting and NF pituitary tumor subtypes, but further investigation is required to better elucidate the extent to which differential methylation exists across subtypes of pituitary tumors (85).

#### Promoter Methylation

The lack of correlation between hypermethylation and gene expression implies that additional regulatory mechanisms beyond methylation remain to be discovered. Methylation and expression levels of the N-myc Downstream-Regulated Gene 2 *(NDRG2)* and Signal Transducer and Activator of Transcription 3 *(STAT3)* promoters are also uncorrelated, along with a lack of correlation with clinical factors (86, 87). Methylation of the Matrix Metallopeptidase 14 *(MMP-14)* and Transforming Growth Factor Beta 1 *(TGF*β*-1)* promoters was also not associated with tumor functionality or recurrence (88). Hypermethylation of the Human Telomerase Reverse Transcriptase *(hTERT*) promoter, which can contribute to cellular immortalization and tumorigenesis, has also been noted across different pituitary tumor subtypes but has not been associated with significant differences in tumor parameters, tumor subtype, or prognosis (89). However, differential methylation of *TERT* promoters has not been consistently observed (90). Larger patient sample sizes are required to better understand the clinical impact of specific epigenetic changes in pituitary tumors.

Methylation at the O^6^-Methylguanine-DNA Methyltransferase *(MGMT)* promoter is of particular interest given its utility epigenetic modification in determining response to temozolomide (TMZ) in glioblastoma (20). Temozolomide has been used for aggressive pituitary adenomas, with mixed results (91, 92). Thus far, *MGMT* expression, rather than promoter methylation, appears to better correlate with TMZ response in pituitary adenomas; however, a limited number of studies have examined this relationship, and TMZ is still administered, particularly in the context of aggressive pituitary tumors, regardless of *MGMT* status (24, 93). MGMT promoter methylation has also been associated with tumor regrowth in pituitary adenoma (94). Even though *MGMT* methylation does not offer as much predictive value for pituitary tumors as glioblastoma, finding clinically informative methylation markers remains the goal.

### Histone Modifications

Acetylation of histone tails, particularly H3 and H4, is generally seen as a mark of active regions of the genome, whereas methylation of histone tails, particularly lysine 9 on H3 (H3K9), is associated with inactive heterochromatin (95). Epigenetic markers can be dynamically modified by chromatin regulators including the histone acetyltransferases (HATs) and histone deacetylases (HDACs).

#### Histone Acetylation Regulators

Multiple chromatin regulators are differentially regulated in pituitary tumors, including *HMGA2* and HDAC2 (73, 78), suggesting that pituitary tumors likely have altered patterns of histone modifications (Table 3). HDAC11 has been shown to interfere with p53 expression in pituitary tumor cells (72). Global acetylation resulted in an increase in Pituitary Tumor-Transforming Gene 1 *(PTTG1)*, Bone Morphogenic Protein 4 *(BMP4)*, and Dopamine Receptor 2 *(DR2)* expression in pituitary tumor cells, suggesting that global alterations in epigenetic modifications may result in gene expression changes (96, 97). Given the nonspecific effects of global acetylation modification, it is unclear how relevant global acetylation findings are to pituitary tumor pathogenesis and therapeutic applications.

The differential expression of some members of the sirtuin family (*SIRT*) of HDACs has been observed in somatotropinomas as compared to NF pituitary adenomas (76). *SIRT1* was overexpressed in somatotropinomas, while *SIRT3, SIRT4*, and *SIRT7* were under-expressed in NF pituitary adenomas. *SIRT1* overexpression correlated with smaller tumor size, while *SIRT3* under-expression correlated with larger tumor size. There was no association between sirtuin levels and invasiveness or Ki-67 proliferative index.

#### Histone Methylation Regulators

Non-invasive pituitary adenomas express significantly higher levels of RIZI, which acts as a tumor-suppressor as well as a possible histone methyltransferase, and lower levels of C-myc, as compared to invasive pituitary adenomas (74). Increased RIZI expression also correlates with significant differences in methylation at four CpG sites, reduced H3K4/H3K9 methylation, and enhanced H3K27 methylation, as well as significantly longer progression-free survival (74). Additionally, p53 mis-expression correlates with H3K9 methylation (98). Specific examples of correlations between epigenetic modifications and gene expression further affirm the possibility that histone modifications may alter gene expression in pituitary tumors.

#### Histone Citrullination Regulators

Peptidylarginine deaminase (PAD) enzymes facilitate histone citrullination, which can modulate chromatin expression (Table 3). Increased PAD prevalence in prolactinomas and somatoprolactinomas has been associated with increased mRNA targeting of oncogenes *HMGA*, Insulin-like Growth Factor 1 *(IGF-1)*, and Neuroblastoma MYC Oncogene *(N-MYC)* by miRNAs, which may yield insight into the etiology of the affected tumor subtypes (75).

## Current Limitations and Future Directions

The limited ability of existing pituitary tumor classification systems to predict clinical behavior motivates investigation into a molecular taxonomy with clinical implications. Though epigenetic signatures are not yet incorporated into clinical decision making for pituitary tumors, the importance of methylation and epigenetic signatures is increasingly appreciated across brain tumors with clinical implications (20, 21). Given the absence of recurrent oncogenic mutations and copy number alterations in many pituitary tumors, epigenetic mechanisms present an intriguing biological avenue for further exploration.

Further elucidation of the mechanisms underlying gene deregulation is needed before viable therapeutic strategies can be developed. Several compounds that inhibit epigenetic modifications are FDA approved, though it remains to be determined whether compounds that globally affect DNA methylation and histone modifications can provide specificity and efficacy in targeting the genetic pathways deregulated in pituitary tumors. More targeted strategies for modulating epigenetic modifications, though currently still in early development, may hold promise for treatment of pituitary tumors (99). Ultimately, an integrated classification of epigenetic, genetic, and histopathologic features may augment the collective predictive power of molecular taxonomy in translating to clinical practice.

## Author Contributions

BH, AL, and SG contributed to the review of the literature and the drafting of the manuscript. WB and ID conceived of the project and provided guidance throughout the writing process.

### Conflict of Interest Statement

The authors declare that the research was conducted in the absence of any commercial or financial relationships that could be construed as a potential conflict of interest.
